# {*N*-[1-(1*H*-Benzimidazol-2-yl)ethyl­idene-κ*N* ^3^]-3-(1*H*-imidazol-1-yl)propan-1-amine-κ*N*}dibromidomercury(II)

**DOI:** 10.1107/S1600536811051166

**Published:** 2011-12-14

**Authors:** Qing Wang, Zhong-Ye Fu, Liang-Min Yu

**Affiliations:** a Education Ministry Key Laboratory of Marine Chemistry and Technology, Ocean University of China, Qingdao, People’s Republic of China

## Abstract

In the title compound, [HgBr_2_(C_15_H_17_N_5_)], the Hg^II^ ion is tetra­hedrally coordinated by two N atoms of the *N*-[1-(1*H*-benzimidazol-2-yl)ethyl­idene-κ*N*]-3-(1*H*-imidazol-1-yl)propan-1-amine ligand, and two bromide anions. Inter­molecular benzimidazole–imidazole N—H⋯N hydrogen bonds link the mol­ecules into helical chains along the *b*-axis direction and C—H⋯Br hydrogen bonds link these chains into layers parallel to the *bc* plane.

## Related literature

For general background to the design and synthesis of coordination polymers, see: Moulton & Zaworotko (2001 ▶); Roesky & Andruh (2003 ▶); Li *et al.* (2007 ▶); Zheng *et al.* (2011 ▶). For complexes with ligands containing benzimidazole or imidazole, see: Pan *et al.* (2010 ▶); Chen *et al.* (2007 ▶); Zhuang *et al.* (2009 ▶); Wang *et al.* (2009 ▶).
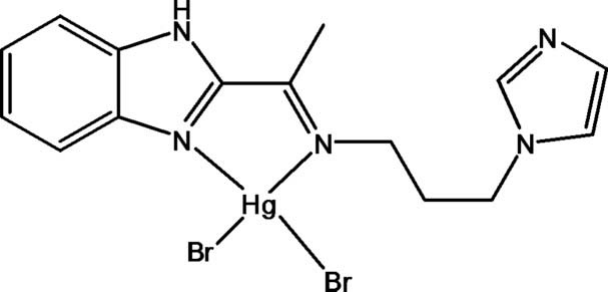


## Experimental

### 

#### Crystal data

[HgBr_2_(C_15_H_17_N_5_)]
*M* *_r_* = 627.75
Monoclinic, 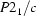

*a* = 10.3054 (4) Å
*b* = 10.6680 (4) Å
*c* = 16.6030 (5) Å
β = 100.844 (3)°
*V* = 1792.71 (11) Å^3^
*Z* = 4
Mo *K*α radiation
μ = 13.05 mm^−1^
*T* = 298 K
0.37 × 0.33 × 0.30 mm

#### Data collection

Bruker SMART APEXII CCD area-detector diffractometer
Absorption correction: multi-scan (*SADABS*; Sheldrick, 1996 ▶) *T*_min_ = 0.086, *T*_max_ = 0.111
9489 measured reflections
3891 independent reflections
2302 reflections with *I* > 2σ(*I*)
*R*_int_ = 0.047

#### Refinement

*R*[*F* ^2^ > 2σ(*F* ^2^)] = 0.031
*wR*(*F* ^2^) = 0.041
*S* = 0.93
3891 reflections
208 parameters
H-atom parameters constrained
Δρ_max_ = 1.45 e Å^−3^
Δρ_min_ = −0.96 e Å^−3^

### 

Data collection: *APEX2* (Bruker, 2004 ▶); cell refinement: *SAINT* (Bruker, 2004 ▶); data reduction: *SAINT* (Bruker, 2004 ▶); program(s) used to solve structure: *SHELXS97* (Sheldrick, 2008 ▶); program(s) used to refine structure: *SHELXL97* (Sheldrick, 2008 ▶); molecular graphics: *XP* in *SHELXTL* (Sheldrick, 2008 ▶); software used to prepare material for publication: *SHELXL97* (Sheldrick, 2008 ▶).

## Supplementary Material

Crystal structure: contains datablock(s) I, global. DOI: 10.1107/S1600536811051166/kp2360sup1.cif

Structure factors: contains datablock(s) I. DOI: 10.1107/S1600536811051166/kp2360Isup2.hkl

Additional supplementary materials:  crystallographic information; 3D view; checkCIF report

## Figures and Tables

**Table 1:** Selected bond lengths (Å)

Hg1—N1	2.274 (4)
Hg1—N3	2.403 (4)
Hg1—Br2	2.4996 (7)
Hg1—Br1	2.5472 (7)

**Table 2:** Hydrogen-bond geometry (Å, °)

*D*—H⋯*A*	*D*—H	H⋯*A*	*D*⋯*A*	*D*—H⋯*A*
N2—H2*B*⋯N5^i^	0.86	1.90	2.722 (7)	160
C15—H15*A*⋯Br1^ii^	0.93	2.85	3.778 (6)	177

Symmetry codes: (i) 
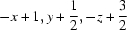
; (ii) 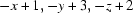.

**Fig. 1.**
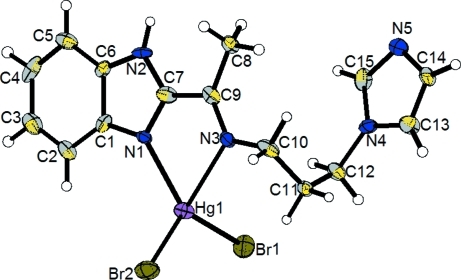
A view of the molecule of (I) showing the atom-labelling scheme. Displacement ellipsoids are drawn at the 30% probability level. H atoms are represented as small spheres of arbitrary radii.

## supplementary crystallographic information

### Comment

The rational design and synthesis of coordination polymers have received extensive attention over the past decades (Moulton & Zaworotko, 2001; Roesky & Andruh, 2003). The choice of suitable ligands is an important factor that greatly affects the structure and stabilisation of the coordination architecture (Zheng *et al.*, 2011). The ligands containing benzimidazole or imidazole groups are often employed to prepare coordination polymers with diverse structure topologies and properties (Pan, *et al.*, 2010; Zhuang, *et al.*, 2009; Chen, *et al.*, 2007). However, incorporation both two functional groups into one ligand are still less explored. Herein we report a Hg^II^ complex with a new ligand containing both benzimidazole and imidazole donor groups, *N*-(1-(1*H*-benzimidazol-2-yl)ethylidene)-3-(1*H*-imidazol-1-yl)propan-1-amine.

The molecule of the title complex is a discrete neutral monomer, in which the asymmetric unit contains one Hg^II^ ion, two Br¯ anions and one ligand (Fig. 1 and Table 1). The Hg^II^ ion has a slightly distorted tetrahedral geometry involving the two nitrogen atoms from the ligand and two Br¯ anions. The bond angles around Hg^II^ are range from 74.20 (14)° to 172.49 (15)°. The Hg—N and Hg—Br bond lengths are 2.274 (4), 2.403 (4) and 2.4996 (7), 2.5472 (7) Å (Table 1), respectively, which is similar to the reported Hg^II^ complexes (Wang *et al.* 2009; Li *et al.* 2007). The nitrogen atom of the imidazolyl group (N_imi_) remains uncoordinated and just acts as a strong hydrogen bonding donor. The N—H···N hydrogen bonds (Table 2) formed between the NH of the benzimidazole group (NH_bim_) and N_im_ link the molecules into helical chain around the crystallographic 2_1_ axis, with the pitches of 8.68 Å. These chains are connected by C—H···Br hydrogen bonds (Table 2) between the carbon atom on 2-position of the imidazole group and Br¯ anion to generate a two-dimensional network.

### Experimental

For general background to the design and synthesis of coordination polymers, see: Moulton & Zaworotko (2001); Roesky & Andruh (2003); Li *et al.* (2007); Zheng *et al.* (2011). For complexes with ligands containing benzimidazole or imidazole group, see: Pan *et al.* (2010); Chen *et al.* (2007); Zhuang, *et al.* (2009); Wang, *et al.* (2009).

### Refinement

All C– and N-bound H atoms were positioned geometrically and refined using a riding model, with C–H = 0.93 Å and N–H = 0.86 Å and with *U*ĩso(H) =1.2*U*eq (C or N).

### Crystal data

**Table:**

[HgBr_2_(C_15_H_17_N_5_)]	*Z* = 4
*M**_r_* = 627.75	*F*(000) = 1168
Monoclinic, *P*2_1_/*c*	*D*_x_ = 2.326 Mg m^−^^3^
Hall symbol: -P 2ybc	Mo *K*α radiation, λ = 0.71073 Å
*a* = 10.3054 (4) Å	θ = 2.8–27°
*b* = 10.6680 (4) Å	µ = 13.05 mm^−^^1^
*c* = 16.6030 (5) Å	*T* = 298 K
β = 100.844 (3)°	Block, colourless
*V* = 1792.71 (11) Å^3^	0.37 × 0.33 × 0.30 mm

### Data collection

**Table:**

Bruker SMART APEXII CCD area-detector diffractometer	3891 independent reflections
Radiation source: fine-focus sealed tube	2302 reflections with *I* > 2σ(*I*)
graphite	*R*_int_ = 0.047
phi and ω scans	θ_max_ = 27.0°, θ_min_ = 2.8°
Absorption correction: multi-scan (*SADABS*; Sheldrick, 1996)	*h* = −13→13
*T*_min_ = 0.086, *T*_max_ = 0.111	*k* = −13→12
9489 measured reflections	*l* = −21→20

### Refinement

**Table:**

Refinement on *F*^2^	Primary atom site location: structure-invariant direct methods
Least-squares matrix: full	Secondary atom site location: difference Fourier map
*R*[*F*^2^ > 2σ(*F*^2^)] = 0.031	Hydrogen site location: inferred from neighbouring sites
*wR*(*F*^2^) = 0.041	H-atom parameters constrained
*S* = 0.93	*w* = 1/[σ^2^(*F*_o_^2^) + (0.*P*)^2^] where *P* = (*F*_o_^2^ + 2*F*_c_^2^)/3
3891 reflections	(Δ/σ)_max_ = 0.003
208 parameters	Δρ_max_ = 1.45 e Å^−^^3^
0 restraints	Δρ_min_ = −0.96 e Å^−^^3^

### Special details

**Table:**

Geometry. All e.s.d.'s (except the e.s.d. in the dihedral angle between two l.s. planes) are estimated using the full covariance matrix. The cell e.s.d.'s are taken into account individually in the estimation of e.s.d.'s in distances, angles and torsion angles; correlations between e.s.d.'s in cell parameters are only used when they are defined by crystal symmetry. An approximate (isotropic) treatment of cell e.s.d.'s is used for estimating e.s.d.'s involving l.s. planes.
Refinement. Refinement of *F*^2^ against ALL reflections. The weighted *R*-factor *wR* and goodness of fit *S* are based on *F*^2^, conventional *R*-factors *R* are based on *F*, with *F* set to zero for negative *F*^2^. The threshold expression of *F*^2^ > σ(*F*^2^) is used only for calculating *R*-factors(gt) *etc*. and is not relevant to the choice of reflections for refinement. *R*-factors based on *F*^2^ are statistically about twice as large as those based on *F*, and *R*- factors based on ALL data will be even larger.

### Fractional atomic coordinates and isotropic or equivalent isotropic displacement parameters (Å^2^)

**Table:**

	*x*	*y*	*z*	*U*_iso_*/*U*_eq_	
Hg1	0.28943 (2)	1.69192 (2)	1.065782 (13)	0.02437 (7)	
Br2	0.09981 (6)	1.80104 (7)	1.10987 (3)	0.03381 (17)	
Br1	0.34657 (6)	1.46862 (6)	1.11412 (3)	0.03254 (18)	
N3	0.2664 (4)	1.6902 (5)	0.9191 (2)	0.0196 (11)	
N1	0.4643 (4)	1.7966 (4)	1.0326 (2)	0.0153 (11)	
C7	0.4677 (5)	1.7955 (6)	0.9526 (3)	0.0220 (14)	
C1	0.5760 (5)	1.8564 (5)	1.0723 (3)	0.0164 (14)	
C6	0.6472 (6)	1.8935 (5)	1.0109 (3)	0.0179 (14)	
C9	0.3613 (6)	1.7408 (5)	0.8910 (3)	0.0176 (14)	
C10	0.1465 (5)	1.6394 (5)	0.8673 (3)	0.0242 (16)	
H10A	0.1617	1.6287	0.8118	0.029*	
H10B	0.0746	1.6986	0.8656	0.029*	
N4	0.2183 (5)	1.3873 (4)	0.8074 (3)	0.0210 (12)	
N2	0.5751 (4)	1.8553 (4)	0.9374 (3)	0.0204 (12)	
H2B	0.5949	1.8674	0.8900	0.025*	
N5	0.3055 (5)	1.3730 (4)	0.6951 (3)	0.0264 (13)	
C2	0.6247 (6)	1.8854 (5)	1.1536 (3)	0.0242 (16)	
H2A	0.5776	1.8646	1.1943	0.029*	
C5	0.7670 (6)	1.9576 (6)	1.0293 (3)	0.0259 (16)	
H5A	0.8123	1.9828	0.9886	0.031*	
C8	0.3749 (5)	1.7525 (5)	0.8032 (3)	0.0225 (15)	
H8A	0.3003	1.7140	0.7687	0.034*	
H8B	0.4545	1.7114	0.7953	0.034*	
H8C	0.3788	1.8395	0.7891	0.034*	
C4	0.8144 (6)	1.9815 (5)	1.1109 (4)	0.0318 (18)	
H4A	0.8950	2.0223	1.1260	0.038*	
C15	0.3258 (6)	1.4068 (5)	0.7735 (3)	0.0252 (16)	
H15A	0.4049	1.4400	0.8017	0.030*	
C14	0.1767 (6)	1.3299 (5)	0.6795 (3)	0.0250 (16)	
H14A	0.1328	1.2997	0.6292	0.030*	
C3	0.7431 (6)	1.9453 (6)	1.1726 (3)	0.0295 (17)	
H3A	0.7782	1.9630	1.2272	0.035*	
C13	0.1234 (6)	1.3376 (5)	0.7478 (3)	0.0272 (17)	
H13A	0.0386	1.3138	0.7531	0.033*	
C11	0.1081 (5)	1.5158 (5)	0.8992 (3)	0.0198 (14)	
H11A	0.0221	1.4915	0.8686	0.024*	
H11B	0.1007	1.5253	0.9562	0.024*	
C12	0.2065 (6)	1.4126 (5)	0.8924 (3)	0.0268 (16)	
H12A	0.2923	1.4364	0.9234	0.032*	
H12B	0.1791	1.3366	0.9165	0.032*	

### Atomic displacement parameters (Å^2^)

**Table:**

	*U*^11^	*U*^22^	*U*^33^	*U*^12^	*U*^13^	*U*^23^
Hg1	0.02425 (14)	0.03019 (14)	0.01982 (12)	0.00036 (16)	0.00710 (9)	0.00116 (13)
Br2	0.0283 (4)	0.0368 (4)	0.0408 (4)	0.0044 (4)	0.0179 (3)	0.0032 (4)
Br1	0.0381 (5)	0.0283 (4)	0.0284 (4)	0.0013 (3)	−0.0008 (3)	0.0050 (3)
N3	0.026 (3)	0.020 (3)	0.014 (2)	0.001 (3)	0.006 (2)	−0.002 (2)
N1	0.019 (3)	0.015 (3)	0.014 (2)	0.003 (3)	0.008 (2)	0.000 (2)
C7	0.021 (4)	0.028 (4)	0.017 (3)	0.004 (3)	0.002 (3)	0.004 (3)
C1	0.013 (4)	0.011 (3)	0.023 (3)	0.003 (3)	−0.003 (3)	0.006 (3)
C6	0.016 (4)	0.013 (4)	0.025 (4)	0.000 (3)	0.005 (3)	0.001 (3)
C9	0.018 (4)	0.016 (3)	0.018 (3)	0.014 (3)	0.002 (3)	0.002 (3)
C10	0.026 (4)	0.035 (4)	0.011 (3)	0.008 (3)	0.001 (3)	0.003 (3)
N4	0.016 (3)	0.026 (3)	0.022 (3)	−0.005 (3)	0.008 (2)	0.000 (2)
N2	0.016 (3)	0.027 (3)	0.021 (3)	0.001 (2)	0.012 (2)	0.004 (2)
N5	0.029 (4)	0.030 (3)	0.021 (3)	−0.003 (3)	0.008 (3)	−0.001 (2)
C2	0.027 (4)	0.026 (4)	0.021 (4)	0.004 (3)	0.006 (3)	−0.005 (3)
C5	0.028 (4)	0.029 (4)	0.022 (3)	0.010 (3)	0.010 (3)	0.004 (3)
C8	0.024 (4)	0.027 (4)	0.017 (3)	0.000 (3)	0.004 (3)	−0.003 (3)
C4	0.020 (4)	0.017 (4)	0.055 (5)	−0.004 (3)	−0.002 (4)	0.006 (3)
C15	0.016 (4)	0.028 (4)	0.027 (4)	−0.002 (3)	−0.006 (3)	−0.004 (3)
C14	0.025 (4)	0.033 (4)	0.016 (3)	−0.008 (3)	0.001 (3)	−0.009 (3)
C3	0.039 (5)	0.027 (4)	0.021 (4)	0.009 (4)	0.002 (3)	0.000 (3)
C13	0.018 (4)	0.041 (5)	0.023 (3)	−0.003 (3)	0.003 (3)	−0.005 (3)
C11	0.017 (4)	0.023 (4)	0.022 (3)	−0.003 (3)	0.009 (3)	−0.007 (3)
C12	0.034 (4)	0.032 (4)	0.014 (3)	−0.007 (3)	0.004 (3)	−0.004 (3)

### Geometric parameters (Å, °)

**Table:**

Hg1—N1	2.274 (4)	N5—C15	1.329 (6)
Hg1—N3	2.403 (4)	N5—C14	1.382 (6)
Hg1—Br2	2.4996 (7)	C2—C3	1.361 (7)
Hg1—Br1	2.5472 (7)	C2—H2A	0.9300
N3—C9	1.280 (6)	C5—C4	1.374 (7)
N3—C10	1.469 (6)	C5—H5A	0.9300
N1—C7	1.335 (5)	C8—H8A	0.9600
N1—C1	1.372 (6)	C8—H8B	0.9600
C7—N2	1.342 (6)	C8—H8C	0.9600
C7—C9	1.472 (7)	C4—C3	1.422 (7)
C1—C2	1.384 (6)	C4—H4A	0.9300
C1—C6	1.420 (7)	C15—H15A	0.9300
C6—N2	1.366 (6)	C14—C13	1.352 (6)
C6—C5	1.393 (7)	C14—H14A	0.9300
C9—C8	1.497 (6)	C3—H3A	0.9300
C10—C11	1.502 (7)	C13—H13A	0.9300
C10—H10A	0.9700	C11—C12	1.514 (7)
C10—H10B	0.9700	C11—H11A	0.9700
N4—C15	1.350 (6)	C11—H11B	0.9700
N4—C13	1.360 (6)	C12—H12A	0.9700
N4—C12	1.465 (6)	C12—H12B	0.9700
N2—H2B	0.8600		
			
N1—Hg1—N3	71.94 (15)	C3—C2—H2A	120.8
N1—Hg1—Br2	122.76 (11)	C1—C2—H2A	120.8
N3—Hg1—Br2	111.53 (11)	C4—C5—C6	116.3 (5)
N1—Hg1—Br1	112.88 (11)	C4—C5—H5A	121.8
N3—Hg1—Br1	106.56 (12)	C6—C5—H5A	121.8
Br2—Hg1—Br1	119.37 (2)	C9—C8—H8A	109.5
C9—N3—C10	124.0 (5)	C9—C8—H8B	109.5
C9—N3—Hg1	115.4 (4)	H8A—C8—H8B	109.5
C10—N3—Hg1	120.5 (3)	C9—C8—H8C	109.5
C7—N1—C1	107.8 (5)	H8A—C8—H8C	109.5
C7—N1—Hg1	114.0 (4)	H8B—C8—H8C	109.5
C1—N1—Hg1	138.0 (3)	C5—C4—C3	121.6 (6)
N1—C7—N2	111.1 (5)	C5—C4—H4A	119.2
N1—C7—C9	122.6 (5)	C3—C4—H4A	119.2
N2—C7—C9	126.2 (5)	N5—C15—N4	112.1 (5)
N1—C1—C2	133.6 (5)	N5—C15—H15A	124.0
N1—C1—C6	106.6 (5)	N4—C15—H15A	124.0
C2—C1—C6	119.8 (5)	C13—C14—N5	110.5 (5)
N2—C6—C5	130.8 (5)	C13—C14—H14A	124.8
N2—C6—C1	106.9 (5)	N5—C14—H14A	124.8
C5—C6—C1	122.4 (5)	C2—C3—C4	121.5 (5)
N3—C9—C7	115.8 (5)	C2—C3—H3A	119.2
N3—C9—C8	127.4 (5)	C4—C3—H3A	119.2
C7—C9—C8	116.8 (5)	C14—C13—N4	106.5 (5)
N3—C10—C11	111.5 (4)	C14—C13—H13A	126.8
N3—C10—H10A	109.3	N4—C13—H13A	126.8
C11—C10—H10A	109.3	C10—C11—C12	112.8 (4)
N3—C10—H10B	109.3	C10—C11—H11A	109.0
C11—C10—H10B	109.3	C12—C11—H11A	109.0
H10A—C10—H10B	108.0	C10—C11—H11B	109.0
C15—N4—C13	107.0 (5)	C12—C11—H11B	109.0
C15—N4—C12	126.6 (5)	H11A—C11—H11B	107.8
C13—N4—C12	126.4 (5)	N4—C12—C11	112.6 (4)
C7—N2—C6	107.7 (4)	N4—C12—H12A	109.1
C7—N2—H2B	126.2	C11—C12—H12A	109.1
C6—N2—H2B	126.2	N4—C12—H12B	109.1
C15—N5—C14	104.0 (5)	C11—C12—H12B	109.1
C3—C2—C1	118.4 (5)	H12A—C12—H12B	107.8
			
N1—Hg1—N3—C9	3.1 (4)	N1—C7—C9—N3	−2.2 (8)
Br2—Hg1—N3—C9	122.0 (4)	N2—C7—C9—N3	−178.6 (5)
Br1—Hg1—N3—C9	−106.1 (4)	N1—C7—C9—C8	176.8 (5)
N1—Hg1—N3—C10	−173.8 (4)	N2—C7—C9—C8	0.4 (8)
Br2—Hg1—N3—C10	−54.9 (4)	C9—N3—C10—C11	137.0 (5)
Br1—Hg1—N3—C10	77.0 (4)	Hg1—N3—C10—C11	−46.3 (5)
N3—Hg1—N1—C7	−4.0 (4)	N1—C7—N2—C6	2.1 (6)
Br2—Hg1—N1—C7	−108.5 (4)	C9—C7—N2—C6	178.8 (5)
Br1—Hg1—N1—C7	96.8 (4)	C5—C6—N2—C7	179.1 (6)
N3—Hg1—N1—C1	−178.7 (6)	C1—C6—N2—C7	−1.4 (6)
Br2—Hg1—N1—C1	76.9 (5)	N1—C1—C2—C3	178.9 (6)
Br1—Hg1—N1—C1	−77.9 (5)	C6—C1—C2—C3	−2.4 (8)
C1—N1—C7—N2	−1.8 (6)	N2—C6—C5—C4	−179.6 (5)
Hg1—N1—C7—N2	−178.1 (4)	C1—C6—C5—C4	1.0 (8)
C1—N1—C7—C9	−178.7 (5)	C6—C5—C4—C3	−1.5 (8)
Hg1—N1—C7—C9	5.1 (7)	C14—N5—C15—N4	0.1 (6)
C7—N1—C1—C2	179.6 (6)	C13—N4—C15—N5	0.3 (7)
Hg1—N1—C1—C2	−5.5 (10)	C12—N4—C15—N5	179.3 (5)
C7—N1—C1—C6	0.8 (6)	C15—N5—C14—C13	−0.4 (6)
Hg1—N1—C1—C6	175.7 (4)	C1—C2—C3—C4	2.0 (9)
N1—C1—C6—N2	0.4 (6)	C5—C4—C3—C2	0.0 (9)
C2—C1—C6—N2	−178.6 (5)	N5—C14—C13—N4	0.6 (6)
N1—C1—C6—C5	179.9 (5)	C15—N4—C13—C14	−0.5 (6)
C2—C1—C6—C5	0.9 (8)	C12—N4—C13—C14	−179.6 (5)
C10—N3—C9—C7	175.0 (5)	N3—C10—C11—C12	−67.2 (6)
Hg1—N3—C9—C7	−1.7 (6)	C15—N4—C12—C11	114.2 (6)
C10—N3—C9—C8	−3.8 (9)	C13—N4—C12—C11	−67.0 (7)
Hg1—N3—C9—C8	179.4 (4)	C10—C11—C12—N4	−61.9 (6)

### Hydrogen-bond geometry (Å, °)

**Table:**

*D*—H···*A*	*D*—H	H···*A*	*D*···*A*	*D*—H···*A*
N2—H2B···N5^i^	0.86	1.90	2.722 (7)	160
C15—H15A···Br1^ii^	0.93	2.85	3.778 (6)	177.

Symmetry codes: (i) −*x*+1, *y*+1/2, −*z*+3/2; (ii) −*x*+1, −*y*+3, −*z*+2.
